# Effects of a combination of bifidobacteria quadruple viable bacteria tablets and quadruple therapy on inflammatory response and *Helicobacter pylori* eradication rate in patients with *Helicobacter pylori* positive gastric ulcers

**DOI:** 10.3389/fmed.2025.1636039

**Published:** 2025-09-29

**Authors:** Chunying Jiang, Jing Shi, Xinfu Zhuang, Meng Zhou, Haiyan Zhong

**Affiliations:** Department of Gastroenterology, Changzhou Tumor Hospital, Changzhou, Jiangsu, China

**Keywords:** gastric ulcer, *Helicobacter pylori*, quadruple therapy, bifidobacteria quadruple viable bacteria tablets, gastrointestinal hormones, inflammatory factors

## Abstract

**Objective:**

To explore the effects of bifidobacteria quadruple viable bacteria tablets (*Bifidobacterium infantis*, *Lactobacillus acidophilus*, *Enterococcus faecalis*, and *Bacillus cereus*) plus quadruple therapy on inflammatory response and *Helicobacter pylori* (*Hp*) eradication rate in patients with *Hp*-positive gastric ulcers.

**Methods:**

One hundred patients with *Hp*-positive gastric ulcers admitted in our hospital from January 2022 to December 2024 were included and divided into a control group and a study group. The former accepted quadruple therapy (esomeprazole magnesium enteric-coated capsules + colloidal bismuth pectin capsule + clarithromycin tablets + amoxicillin capsule). Based on the quadruple therapy, the latter was added with bifidobacteria quadruple viable bacteria tablets. The clinical symptoms, clinical efficacy, *Hp* eradication rate, levels of gastrointestinal hormones and inflammatory factors, immune function, number of beneficial bacteria, and incidence of adverse reactions were compared in both groups.

**Results:**

Following 2 weeks of treatment, the study group had lower scores across these symptoms compared to the control group (*P* < 0.05). Furthermore, the study group had a higher total treatment efficacy rate and a higher *Hp* eradication rate compared to the control group (*P* < 0.05). Compared to the control group, the study group had higher levels of serum somatostatin and lower levels of motilin, gastrin, and pepsinogen I following 2 weeks of treatment (*P* < 0.05). Additionally, the study group had lower levels of interleukin-6 (IL-6), C-reactive protein (CRP), interleukin-8 (IL-8), and matrix metalloproteinase-9 (MMP-9) compared to the control group (*P* < 0.05). In comparison to the control group, the study group had higher levels of CD4 + T-cells, higher CD4 + /CD8 + ratio and lower CD8 + T-cell levels after 2 weeks of treatment (*P* < 0.05). Moreover, the study group had a significant increase in the abundance of beneficial gut microbiota, specifically *Enterococcus faecalis*, *Lactobacillus acidophilus*, and *Bifidobacterium* compared to the control group (*P* < 0.05). Lastly, the study group had a lower incidence of adverse reactions than the control group (*P* < 0.05).

**Conclusion:**

Bifidobacteria quadruple viable bacteria tablets plus quadruple therapy can improve the clinical symptoms, promote the clinical therapeutic effect and *Hp* eradication rate, improve the levels of gastrointestinal hormones and inflammatory factors, enhance the immune function, increase levels of beneficial bacteria and diminish the incidence of adverse reactions caused by quadruple therapy in patients suffered from *Hp*-positive gastric ulcer.

## Introduction

Gastric ulcers are a common clinical digestive disease, mainly caused by excessive gastric acid secretion. This excessive secretion can damage the gastric mucosa, resulting in ulcers of varying degrees in the antrum and cardia regions of the stomach (1). Gastric ulcers are closely related to *Helicobacter pylori* (*Hp*) infection (2). *Hp* is the pathogenic bacterium that causes gastric ulcers that can grow and reproduce in acidic environments (3, 4). When the human body is infected with *Hp*, it will enter the stomach, penetrate the mucosal layer, and subsequently damage the function of the gastric mucosa, thereby causing gastric ulcers (5). It has been reported 67.0%–80.0% of gastric ulcers are caused by *Hp* infection (6). If an *Hp*-positive gastric ulcers cannot be treated promptly and effectively, the long-term parasitism of *Hp* in the body may lead to gastrointestinal tumors, thereby endangering the patient’s life (7, 8). Therefore, the eradication of *Hp* plays a key role in the treatment of gastric ulcers.

The main purpose of clinical treatment for *Hp*-positive gastric ulcers is to eradicate *Hp* and alleviate clinical symptoms (9). Quadruple therapy, the first choice for treating *Hp*-positive gastric ulcers, aims to achieve treatment goals through measures such as killing the bacteria, reducing acid production, and protecting the gastric mucosa (10). However, long-term use of this treatment method may lead to the development of drug resistance, thereby reducing its effectiveness in eradicating *Hp*. Moreover, it also disrupts the balance of the intestinal microbiota, thereby hindering the recovery of normal stomach functions (11).

Toll-like receptors (TLRs) play a significant role in the pathogenesis of *Hp*-positive gastric ulcers (12, 13). After *Hp* infection, TLRs on the gastric mucosal epithelial cells and immune cells recognize the pathogen-associated molecular patterns of the bacteria, activating downstream signaling pathways, leading to the activation of transcription factors such as nuclear factor kappaB (NF-κB), thereby mediating the transcription and expression of nuclear inflammatory-related genes (14, 15). This process triggers the release of a large amount of inflammatory cytokines, such as tumor necrosis factor-α (TNF-α), interleukin-1β (IL-1β), and interleukin-6 (IL-6), leading to the formation of an inflammatory cascade and disruption of the immune function, thereby causing damage to the gastric mucosa and the formation of ulcers (16).

Studies have shown that the development of various human diseases is closely linked to an imbalance in the microbial flora (17, 18). Probiotics have gained increasing attention because of their non-toxicity, lack of side effects, and absence of residues. They are now being utilized to regulate the balance of normal microbial flora (19). There is a close regulatory relationship between probiotics and inflammation. Probiotics exert anti-inflammatory effects through various mechanisms, such as regulating intestinal microbiota, strengthening the immune barrier, inhibiting harmful bacteria, and regulating cytokines (18). Studies have shown that probiotics adjuvant therapy can efficiently promote the eradication of *Hp*, inhibit the growth of *Hp*, increase intestinal probiotics, promote intestinal peristalsis, and improve local immunity of the gastrointestinal tract (20, 21). Bifidobacteria quadruple viable bacteria tablets have been widely used in clinical practice in recent years, and have a good effect on chronic diarrhea, antibiotic-resistant constipation, and diarrhea resulting from intestinal flora imbalance (22). It contains *Bifidobacterium infantis*, *Lactobacillus acidophilus*, *Enterococcus faecalis*, and *Bacillus cereus*, which are normal intestinal flora in the body of healthy people and can multiply and grow rapidly in the intestines (23). Relevant studies have found that bifidobacteria quadruple viable bacteria tablets can adjust the balance of intestinal flora, directly supplement the normal physiological flora of the body, and inhibit and kill potentially harmful bacteria in the intestine (24). However, the clinical efficacy of bifidobacteria quadruple viable bacteria tablets plus quadruple therapy in patients with *Hp*-positive gastric ulcers remains largely unclear.

Therefore, our study aimed to assess the effects of bifidobacteria quadruple viable bacteria tablets plus quadruple therapy on inflammatory response and *Hp* eradication rate in patients with *Hp*-positive gastric ulcer.

### Data and methods

#### General data

**FIGURE 1:**
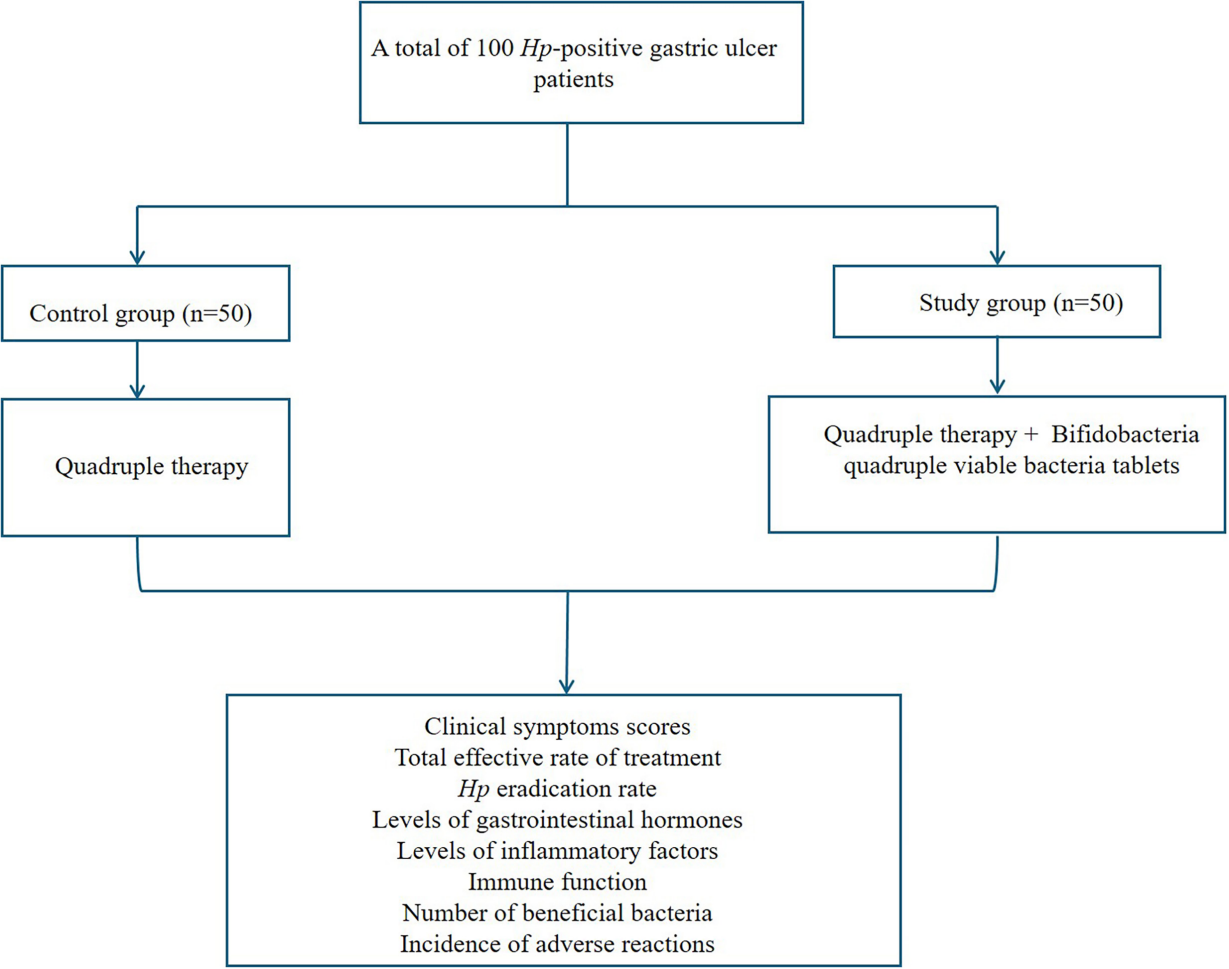
The method process carried out in the current study.

One hundred patients with *Hp*-positive gastric ulcers in our hospital from January 2022 to December 2024 were included and divided into a control group and a study group using the random number table method, with 50 patients in each group. The flowchart of the method process used in the current study was shown in Figure 1. Inclusion criteria: (1) Gastric ulcer was diagnosed by clinical symptoms and gastroscopy; (2) ^14^C urea breath test was *Hp* positive (25); (3) Age ≥ 18 years old. Exclusion criteria: (1) Use of antibiotics, probiotics, bismuth and other drugs in the past 2 weeks; (2) Recent history of gastrointestinal surgery; (3) Allergic to the study drugs; (4) Gastric malignant tumor, gastrointestinal perforation or gastrointestinal bleeding.

### Randomization and blinding

A group randomization design was adopted for random grouping. The random allocation sequence was generated by a computer. The allocation confidentiality measures were achieved through sequential numbering, sealing, and opaque envelopes. After being deemed to meet the inclusion criteria, patients were randomly assigned to the control group or the study group in a 1:1 ratio. This study was single-blind, and the participants were unaware of the allocation.

## Materials and methods

The control group was treated with quadruple therapy based on local *Hp* resistance patterns, including:

(1)    Esomeprazole magnesium enteric-coated capsules (Chia Tai Tianqing Pharmaceutical Group Co., Ltd.; Specification: 20 mg/pill), oral before meals, 20 mg/time, twice a day.
(2)    Colloidal bismuth pectin capsule (Hunan Dibo Pharmaceutical Co., Ltd.; Specification: 50 mg), 0.2 g/time, twice a day.
(3)    Clarithromycin tablets (Changchun Lei Yun Shang Pharmaceutical Co., Ltd.; Specification: 0.25 g/tablet), oral after meals, 0.5 g/time, twice a day.
(4)    Amoxicillin capsule (Sichuan Emeishan Pharmaceutical Co., Ltd.; Specification: 0.5 g), 1.0 g/time, twice a day.

Based on the quadruple therapy, the study group was added with bifidobacteria quadruple viable bacteria tablets (Hangzhou Yuanda Bio-Pharmaceutical Co., Ltd.; Specification: 0.5 g/tablet), 1.5 g/time, 3 times a day.

Both groups received treatment for 2 weeks as described previously (26).

At 4 weeks after treatment, we performed a ^14^C-urea breath test to evaluate *Hp* eradication.

### Observation indicators

(1)    The clinical symptoms including abdominal distension, acid reflux, belching and abdominal pain were scored as follows: (1) 0 point: no symptoms; (2) 1 point: the symptoms were mild and did not affect daily life, and the attack occurred once more than 5 days; (3) 2 points: the symptoms were slightly severe, the effect on daily life was slight, and the attack occurred once more than 3–5 days; (4) 3 points: severe symptoms, seriously affecting daily life, and the attack occurred every other day or every day. Scores for each symptom ranged from 0 to 3, with higher scores representing more severe symptoms.
(2)    After 2 weeks of treatment, the total effective rate of was evaluated as described previously (27). The total effective rate of treatment refers to the percentage of patients whose condition has improved to a certain extent (including obviously effective, effective, and ineffective) after a certain treatment among all patients who received that treatment. It reflects the overall effectiveness of the treatment plan for most patients and is a comprehensive indicator for measuring the treatment outcome. (1) Obviously effective: abdominal distension, acid reflux and other symptoms disappeared, no mucosal edema was found, gastroscopy showed that ulcers disappeared or scar formed, and the clinical symptom score decreased by ≥80.0%. (2) Effective: Gastroscopy showed that the ulcer basically disappeared, with mild edema or inflammation. The clinical symptom score was reduced by 50.0%–79.0%. (3) Ineffective: patients did not meet the criteria of obviously effective or effective. Total effective rate = (Number of obviously effective + number of effective)/total number of cases × 100%.
(3)    After 2 weeks of treatment, *Hp* was determined to be eradicated if the ^14^C urea breath test was negative, and eradication failure if the ^14^C urea breath test was positive.
(4)    Before and following 2 weeks of treatment, 3 mL of venous blood was obtained from the patients, and the serum was collected by centrifugation. The serum levels of somatostatin (SS), motilin (MTL), pepsinogen I (PG I) and gastrin (GAS) were determined by immunoturbidimetry.
(5)    Before and following 2 weeks of treatment, 3 mL of venous blood was obtained from the patients, and the serum was collected by centrifugation. The serum levels of C-reactive protein (CRP), interleukin-6 (IL-6), matrix metalloproteinase-9 (MMP-9) and interleukin-8 (IL-8) levels were measured by enzyme-linked immunosorbent assay (ELISA).
(6)    Before and after 2 weeks of treatment, 3 mL of venous blood was obtained from the patients, and the serum was collected by centrifugation. Flow cytometry (Beckman Coulter Co., Ltd., USA) was used to detect cluster of differentiation 4 positive (CD4^+^) and cluster of differentiation 8 positive (CD8^+^), followed by calculation of CD4^+^/CD8^+^.
(7)    Fresh stool samples were collected from the patients, 1 g and 9 mL glycerol were diluted 10-fold to 1 × 10^8^, and inoculated and cultured. *Enterococcus faecalis* was cultured in an ordinary incubator at 37 °C for 24 h, *Lactobacillus acidophilus* and *Bifidobacterium* were cultured in an anaerobic incubator for 45 h, and the number of bacteria in each gram stool was calculated by l g colony-forming unit (CFU)/g.
(8)    The total incidence of adverse reactions containing dizziness, vomiting, nausea along with diarrhea were calculated.

### Statistical analysis

GraphPad Prism 10.0 statistical software was employed for analyzing the data. The measurement data were exhibited by mean ± standard deviation (x ± s), and *t*-test was conducted for comparison. The counting data were exhibited as number and rate (%), and χ^2^ test was applied for comparison. *P* < 0.05 was considered as statistically significant.

## Results

**FIGURE 2:**
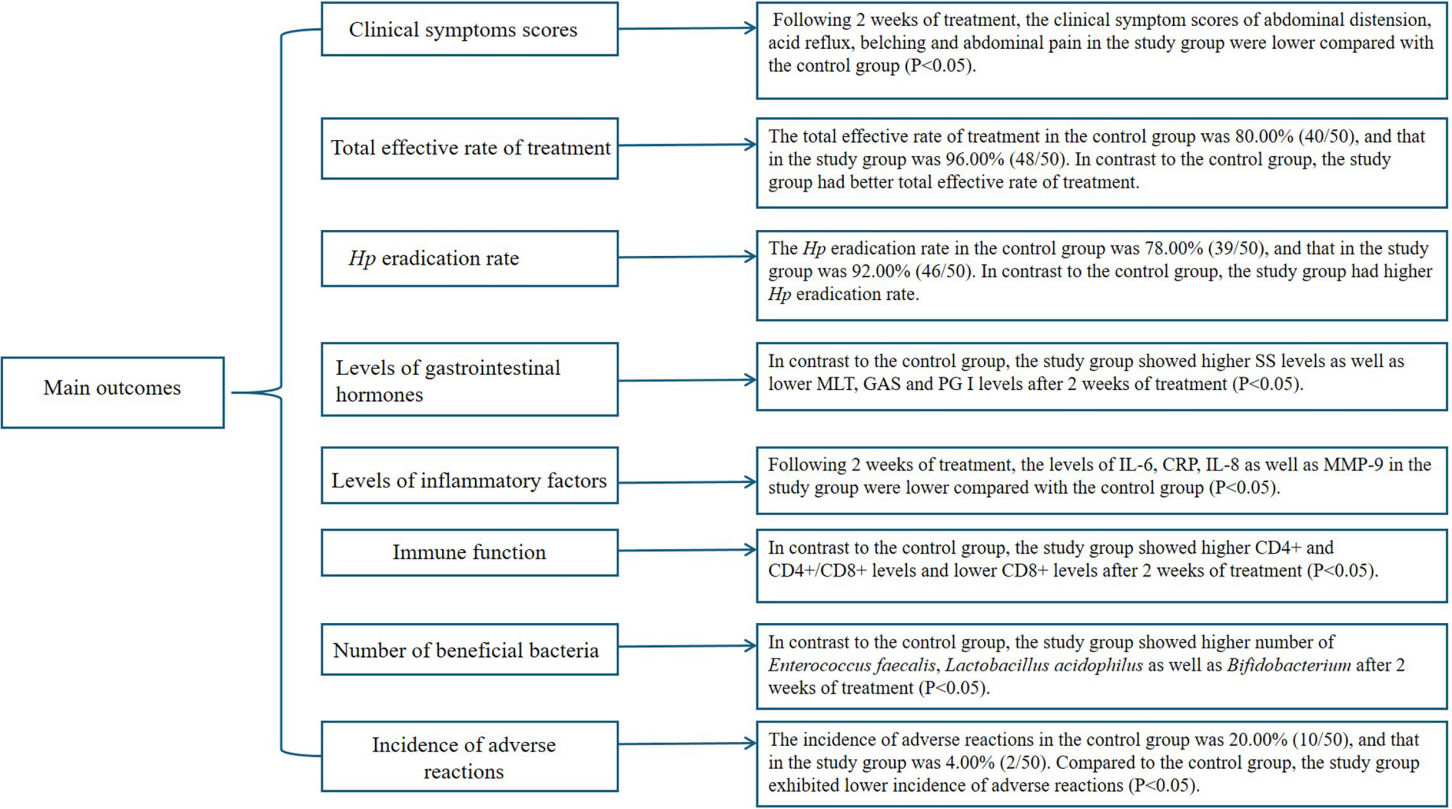
The main outcomes of the study.

The flowchart of the results was shown in Figure 2.

### Patients’ general information in both groups

As Table 1 displayed, no difference was seen in general information containing gender, age, course of disease, diameter of ulcer, location of ulcer and BMI of patients between both groups (*P* > 0.05).

**TABLE 1:** General information of patients in both groups.

Items	Control group (*n* = 50)	Study group (*n* = 50)	χ^2^/t	*P*
Gender			0.36	0.54
Male	28 (56.00)	25 (50.00)		
Female	22 (44.00)	25 (50.00)		
Age (years)	46.04 ± 6.07	46.10 ± 6.15	0.04	0.96
Course of disease (months)	11.72 ± 2.59	11.76 ± 2.63	0.07	0.93
Diameter of ulcer (cm)	1.47 ± 0.30	1.52 ± 0.33	0.79	0.42
Location of ulcer			0.50	0.77
Fundus of stomach	21 (42.00)	20 (40.00)		
Antrum of stomach	18 (36.00)	16 (32.00)		
Body of stomach	11 (22.00)	14 (28.00)		
BMI (kg/m^2^)	22.06 ± 1.98	22.18 ± 2.03	0.29	0.76

### Clinical symptom scores in both groups

**FIGURE 3:**
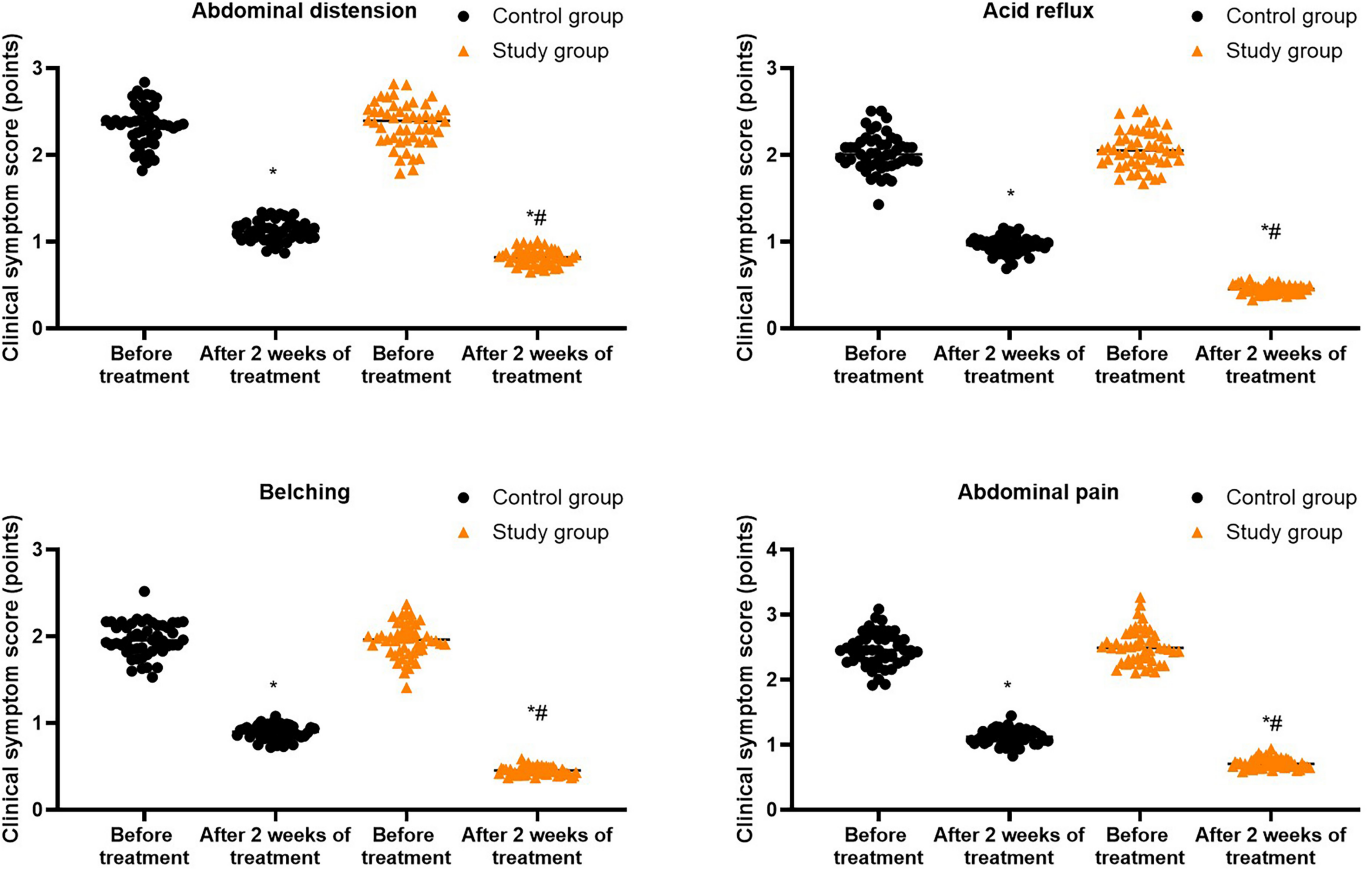
Clinical symptom scores in both groups. *P < 0.05 vs. before treatment; ^#^*P* < 0.05 vs. control group.

Prior to treatment, no differences were seen in the clinical symptom scores of abdominal distension, acid reflux, belching and abdominal pain between both groups (*P* > 0.05). Following 2 weeks of treatment, the clinical symptom scores of abdominal distension, acid reflux, belching and abdominal pain were diminished in both groups (*P* < 0.05), and those in the study group were lower compared with the control group (*P* < 0.05, Figure 3, and Supplementary Table 1).

### Total effective rate of treatment in both groups

As Table 2 revealed, the total effective rate of treatment in the control group was 80.00% (40/50), and that in the study group was 96.00% (48/50). In contrast to the control group, the study group had better total effective rate of treatment (*P* < 0.05).

**TABLE 2:** Total effective rate of treatment in both groups.

Groups	Cases	Obviously effective	Effective	Ineffective	Total effective rate
Control group	50	20 (40.00)	21 (42.00)	9 (18.00)	41 (82.00)
Study group	50	23 (46.00)	25 (50.00)	2 (4.00)	48 (96.00)
χ^2^					5.00
*P*					0.02

### *Hp* eradication rate in both groups

As Table 3 revealed, the *Hp* eradication rate in the control group was 78.00% (39/50), and that in the study group was 92.00% (46/50). In contrast to the control group, the study group had higher *Hp* eradication rate (*P* < 0.05).

**TABLE 3:** *Hp* eradication rate in both groups.

Groups	Cases	Number of *Hp* eradication	*Hp* eradication rate (%)
Control group	50	39	78.00
Study group	50	46	92.00
χ^2^			3.84
*P*			0.04

### Gastrointestinal hormones in both groups

**FIGURE 4:**
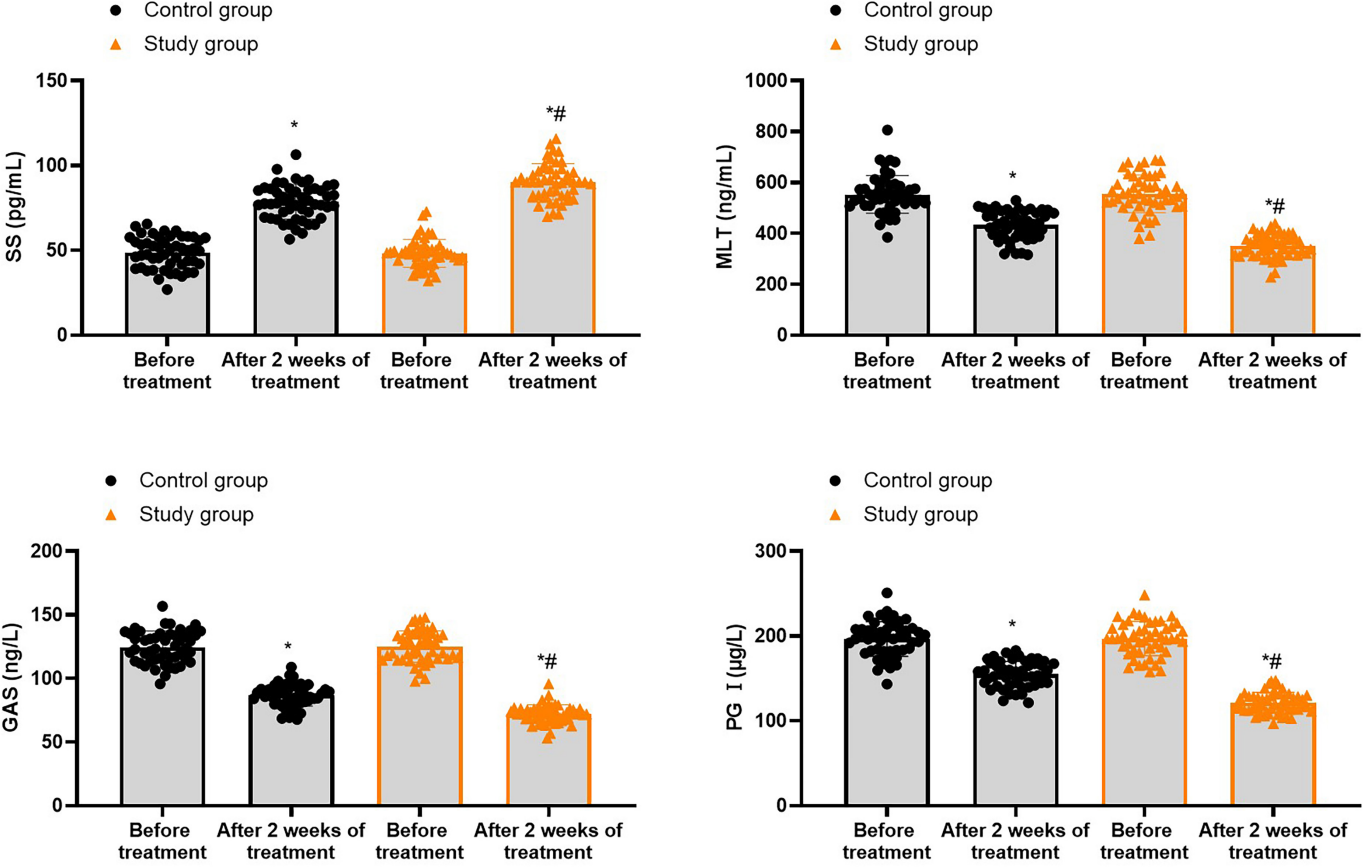
Gastrointestinal hormones in both groups. *P < 0.05 vs. before treatment; ^#^*P* < 0.05 vs. control group.

Prior to treatment, no differences were seen in SS, MLT, GAS and PG I levels between both groups (*P* > 0.05). Following 2 weeks of treatment, the SS levels were elevated while MLT, GAS and PG I levels were declined in both groups (*P* < 0.05). In contrast to the control group, the study group showed higher SS levels as well as lower MLT, GAS and PG I levels 2 weeks of treatment (*P* < 0.05, Figure 4, and Supplementary Table 2).

### Inflammatory response in both groups

**FIGURE 5:**
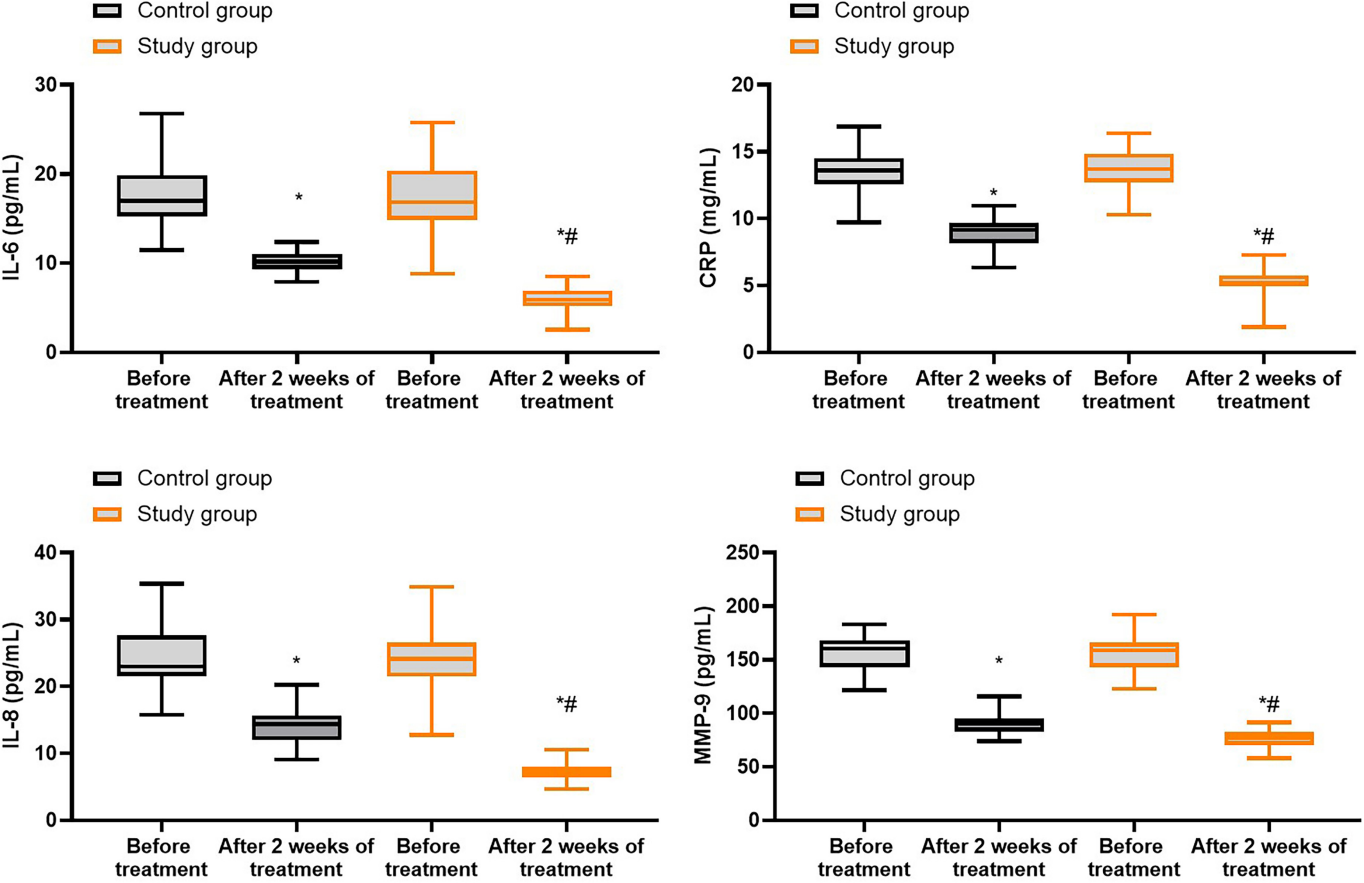
Inflammatory response in both groups. *P < 0.05 vs. before treatment; ^#^*P* < 0.05 vs. control group.

Prior to treatment, no differences were seen in the levels of IL-6, CRP, IL-8 as well as MMP-9 between both groups (*P* > 0.05). Following 2 weeks of treatment, the levels of IL-6, CRP, IL-8 as well as MMP-9 were declined in both groups (*P* < 0.05), and those in the study group were lower compared with the control group (*P* < 0.05, Figure 5, and Supplementary Table 3).

### Immune function in both groups

**FIGURE 6:**
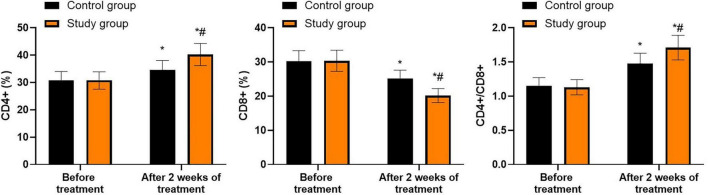
Immune function in both groups. *P < 0.05 vs. before treatment; ^#^*P* < 0.05 vs. control group.

Prior to treatment, no differences were seen in CD4^+^, CD8^+^ and CD4^+^/CD8^+^ levels between both groups (*P* > 0.05). Following 2 weeks of treatment, the CD4^+^ and CD4^+^/CD8^+^ levels were elevated while CD8^+^ levels were declined in both groups (*P* < 0.05). In contrast to the control group, the study group showed higher CD4^+^ and CD4^+^/CD8^+^ levels and lower CD8^+^ levels following 2 weeks of treatment (*P* < 0.05, Figure 6, and Supplementary Table 4).

### Number of beneficial bacteria in both groups

**FIGURE 7:**
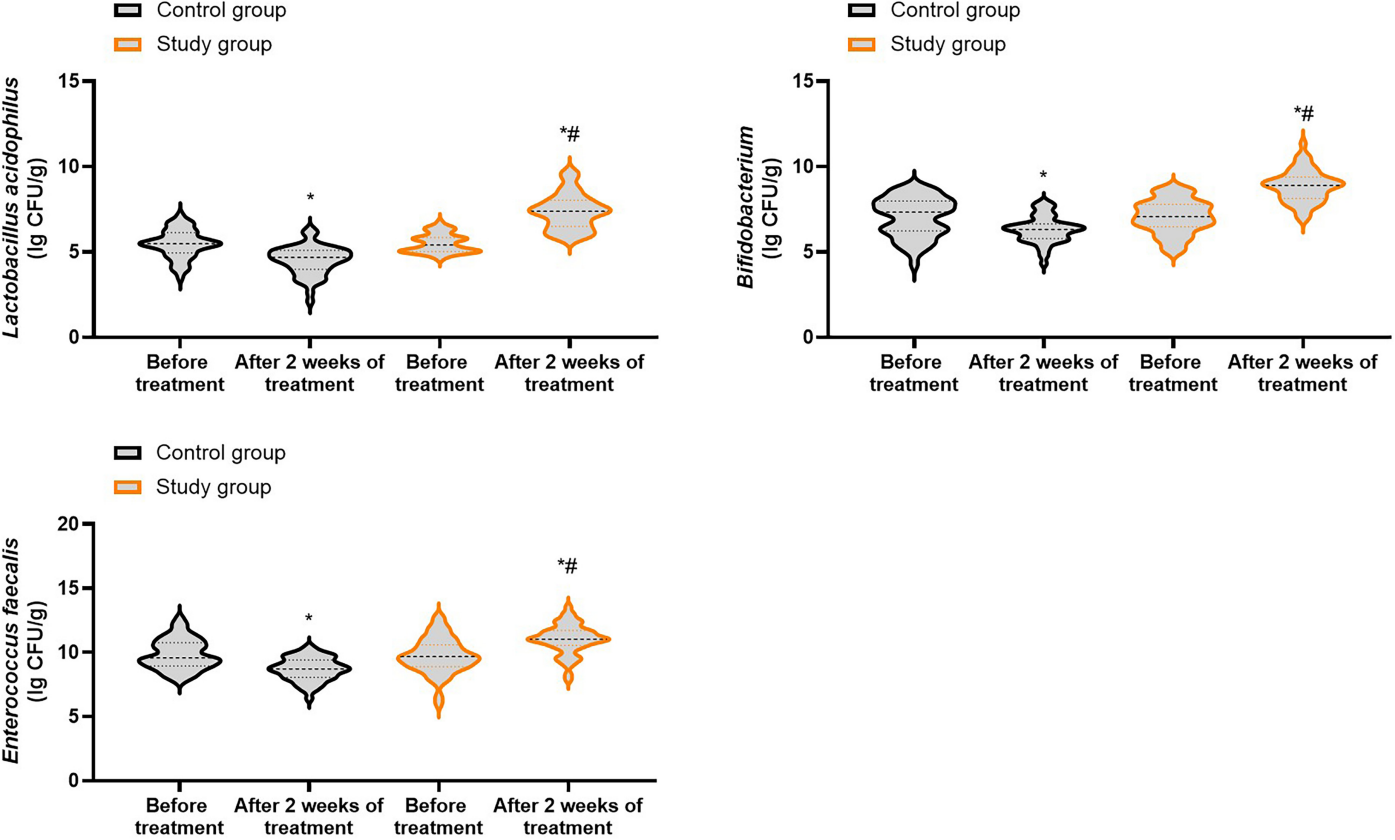
Number of beneficial bacteria in both groups. *P < 0.05 vs. before treatment; ^#^*P* < 0.05 vs. control group.

Prior to treatment, no differences were seen in the number of *Enterococcus faecalis*, *Lactobacillus acidophilus* as well as *Bifidobacterium* between both groups (*P* > 0.05). Following 2 weeks of treatment, the number of *Enterococcus faecalis*, *Lactobacillus acidophilus* and *Bifidobacterium* were elevated in the study group, and those were diminished in the control group (*P* < 0.05). In contrast to the control group, the study group had higher number of *Enterococcus faecalis*, *Lactobacillus acidophilus* and *Bifidobacterium* after 2 weeks of treatment (*P* < 0.05, Figure 7, and Supplementary Table 5).

### Incidence of adverse reactions in both groups

As Table 4 revealed, the incidence of adverse reactions in the control group was 20.00% (10/50), and that in the study group was 4.00% (2/50). Compared to the control group, the study group had lower incidence of adverse reactions (*P* < 0.05).

**TABLE 4:** Incidence of adverse reactions in both groups.

Groups	Cases	Dizziness	Vomiting	Nausea	Diarrhea	Total incidence rate
Control group	50	2 (4.00)	2 (4.00)	4 (8.00)	2 (4.00)	10 (20.00)
Study group	50	0 (0.00)	1 (2.00)	1 (2.00)	0 (0.00)	2 (4.00)
χ^2^						6.06
*P*						0.01

## Discussion

*Hp*-positive gastric ulcers are characterized by gastric mucosa damage. The main cause is that the urease produced by *Hp* acts on the urea surrounding the gastric mucosa, leading to the decomposition of urea and thereby significantly increasing the ammonia concentration and *Hp* content in the gastric juice (28). In addition, *Hp* promotes pepsin and gastric acid secretion. Excessive secretion of these substances increases various factors that damage the gastric mucosa (29). If *Hp*-positive gastric ulcers are not treated promptly, they may damage the gastric mucosa and lead to atrophic gastritis (30). In severe cases, it may develop into gastric cancer, which poses a serious threat to life (31). Therefore, for patients with *Hp*-positive gastric ulcers, active and effective treatment measures are crucial for alleviating symptoms and reducing adverse reactions.

Quadruple therapy is the preferred treatment for *Hp*-positive gastric ulcers (32). However, the quadruple therapy involves the combined use of multiple antibiotics. This multi-drug combination approach may accelerate the development of bacterial resistance, thereby reducing the eradication effect of *Hp* and potentially causing an imbalance in the intestinal flora, which may adversely affect the recovery of gastric function (33). In recent years, the concept of “treating bacteria by bacteria” had provided a new direction for treating *Hp*-positive gastric ulcers (34). The combination of probiotics and antibiotics can not only enhance the therapeutic effect of antibiotics but also help maintain the balance of gastrointestinal flora (35).

In our study, we first compared the general data of patients between the two groups and found no difference in general information, including gender, age, course of disease, ulcer diameter, ulcer location and BMI of patients between both groups, suggesting that the general data of the two groups of patients were comparable.

Patients with *Hp*-positive gastric ulcers may experience clinical symptoms such as abdominal distension and pain, acid reflux, belching, and abdominal pain, which can impact their health (36). The results of this study showed that, compared to the control group, the study group had lower clinical symptom scores for abdominal distension, acid reflux, belching and abdominal pain, a higher total effective rate of treatment, and a higher *Hp* eradication rate following 2 weeks of treatment. These results suggested that bifidobacteria quadruple viable bacteria tablets plus quadruple therapy could improve clinical symptoms, facilitate the clinical therapeutic effect, and enhance the *Hp* eradication rate in patients with *Hp*-positive gastric ulcers. This is because bifidobacteria quadruple viable bacteria tablets can directly supplement the microbial flora on the mucosal surface of the host, improve the imbalance of the microecological environment, change the growth environment of *Hp*, fundamentally block the growth of *Hp*, increase the *Hp* eradication rate, and improve the clinical symptoms and treatment effect when combined with quadruple therapy. Consistently, Zhou et al. (37) suggested that Bifidobacterium quadruple viable tablets combined with quadruple therapy had good efficacy in *Hp*-associated peptic ulcer disease and could promote *Hp* clearance.

Gastrointestinal hormone levels can reflect the severity of gastric ulcers to a certain extent. Among them, SS can inhibit the release of gastrin (38). MTL can not only regulate gastrointestinal motility but also promote gastric acid secretion, thereby stimulating the smooth muscles of the gastrointestinal tract (39). At the same time, *Hp* infection can damage the gastric mucosa, causing monocytes and neutrophils in the gastric antrum to infiltrate the gastric mucosa, stimulating the secretion of pepsin (40). During this process, the content of PG I in the serum increases significantly, which aggravates the erosion of gastric mucosa and may also have a regulatory effect on the secretion of gastrointestinal hormones (41). Furthermore, some studies have found that TLRs are closely related to the occurrence and development of gastric ulcers. They can recognize the pathogen-associated molecular patterns of *Hp*, thereby activating the immune response and exacerbating inflammation (42). After TLRs are activated, they trigger the synthesis and secretion of inflammatory factors through pathways such as the NF-κB pathway. These inflammatory factors further amplify the immune response, forming a positive feedback loop (43). MMP-9 plays a crucial role in tissue healing. When ulcers occur, MMP-9 levels significantly increases, and pro-inflammatory factors such as IL-6 and IL-8 also abnormally increase (44, 45). The results of this study revealed that compared to the control group, the study group had higher SS levels, lower MLT, GAS and PG I levels, and lower IL-6, CRP, IL-8 and MMP-9 levels 2 weeks after treatment. These findings indicated that bifidobacteria quadruple viable bacteria tablets plus quadruple therapy could improve the levels of gastrointestinal hormones and inflammatory factors in patients with *Hp*-positive gastric ulcers. This is because the normal human gut flora contained in bifidobacteria quadruple viable bacteria tablets, such as *Enterococcus* and *Bifidobacterium*, can regulate the harmful bacteria in the gut, thereby maintaining the microecological balance and reducing inflammation of the gastric mucosa (46). In addition, bifidobacteria quadruple viable bacteria tablets have anti-inflammatory and anti-oxidant properties, which can effectively promote the secretion of mucin, thereby stabilizing the mucosal barrier function (47). Moreover, bifidobacteria quadruple viable bacteria tablets can regulate the secretion of gastrointestinal hormones, further promoting the repair process of the gastric mucosa (48). More importantly, bifidobacteria quadruple viable bacteria tablets plus quadruple therapy may improve the levels of gastrointestinal hormones and inflammatory factors in *Hp*-positive gastric ulcers through regulating that TLR/NF-κB signaling pathway. The probiotics in bifidobacteria quadruple viable bacteria tablets bind to the surface components (such as peptidoglycan, lipoteichoic acid) or metabolites (such as short-chain fatty acids, bacteriocins) of the host intestinal epithelial cells or immune cells’ surface TLR2/TLR4, thereby activating the NF-κB signaling pathway (49). Consistently, Niu et al. indicated that probiotic supplementation reduced the levels of inflammatory markers in the body, alleviated the adverse symptoms and gut microbiota disturbances caused by eradication therapy, providing a possible way to improve the overall health of patients (50).

The findings of this study also demonstrated that, compared to the control group, the study group showed higher CD4^+^ and CD4^+^/CD8^+^ levels, lower CD8^+^ levels, and a higher number of *Enterococcus faecalis*, *Lactobacillus acidophilus*, and *Bifidobacterium* 2 weeks after treatment. These results indicated that bifidobacteria quadruple viable bacteria tablets plus quadruple therapy could enhance the immune function and increase levels of beneficial bacteria of patients with *Hp*-positive gastric ulcers. We suggest that this is because a variety of beneficial bifidobacteria quadruple viable bacteria tablets directly supplement the gastrointestinal flora, thereby regulating the microecological balance and heightening patients’ immune function (51). Similarly, Chen et al. suggested that probiotic supplementation could relieve more gastrointestinal symptoms by inducing alterations in gut microbiota and host immune responses (52).

In addition, our study indicated that, compared to the control group, the study group had a lower incidence of adverse reactions, suggesting that bifidobacteria quadruple viable bacteria tablets plus quadruple therapy could diminish the incidence of adverse reactions caused by quadruple therapy in patients with *Hp*-positive gastric ulcers. This is because bifidobacteria quadruple viable bacteria tablets can regulate and balance the gastrointestinal flora of patients, improve the internal environment, and reduce the risk of antibiotic-induced adverse reactions. Jiang et al. conducted a comprehensive and methodical systematic review coupled with a meta-analysis, consistently demonstrating that the occurrence rate of adverse reactions was notably lower when compared to bismuth-based quadruple therapy (53).

Compared with other similar studies, this study further explored the effects of the combined treatment on the levels of gastrointestinal hormones and inflammatory factors. Gastrointestinal hormones play a crucial role in regulating gastrointestinal functions, and inflammatory factors are closely related to the onset and progression of gastric ulcers. By observing the changes in these indicators, we can gain a more comprehensive understanding of the mechanism of the combined treatment and provide a more solid theoretical basis for clinical application. In addition, this study not only focused on the eradication rate of *Hp* and the improvement of clinical symptoms, but also conducted a comprehensive assessment of the patients’ immune function and beneficial bacteria. The enhancement of immune function indicates that the patient’s body’s resistance to pathogens has increased, which is conducive to preventing the recurrence of the disease; while the increased levels of beneficial bacteria reflects the positive impact of this therapy on the balance of intestinal microecology. Currently, although some studies have mentioned that combined treatment has a certain effect on intestinal flora, our research adopted more advanced detection techniques to conduct a more precise analysis of the types and quantities of beneficial bacteria, clarifying which beneficial bacteria have increased, providing more detailed information for optimizing the treatment plan. In summary, the results of this study are expected to have a positive impact on the clinical treatment guidelines for *Hp*-positive gastric ulcers. As our understanding of the mechanism of the combined treatment and the optimization of the treatment plan deepen, this therapy is likely to be more frequently recommended as the first-line treatment option. At the same time, our research also provides new evidence and support for the update and improvement of clinical guidelines, which is conducive to promoting the standardization and development of clinical practice in this field.

### Strengths and limitations

The strength of the study is that it includes multiple observation indicators related to clinical therapeutic effect of *Hp*-positive gastric ulcer. Our research has some limitations. Firstly, our sample size is relatively small, which may lead to deviations between the data results and the actual values. Secondly, our research was a single-center study, and the sample was not representative, which may not accurately reflect the characteristics of a broader population. Fourthly, the observation period for our study was only 2 weeks, and no longer-term follow-up observations were conducted. Therefore, more multi-center, large-scale, and long-term studies should be conducted in the future to further verify our findings.

## Conclusion

Our study demonstrates that bifidobacteria quadruple viable bacteria tablets plus quadruple therapy can improve the clinical symptoms, promote the clinical therapeutic effect and *Hp* eradication rate, improve the levels of gastrointestinal hormones and inflammatory factors, enhance the immune function, increase levels of beneficial bacteria and diminish the incidence of adverse reactions caused by quadruple therapy in patients suffered from *Hp*-positive gastric ulcer.

## Data Availability

The datasets presented in this study can be found in online repositories. The names of the repository/repositories and accession number(s) can be found in this article/Supplementary material.
